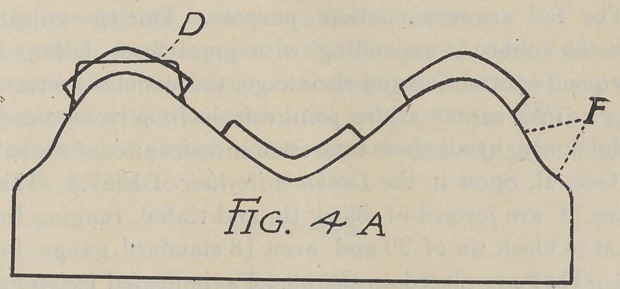# The Reason Why

**Published:** 1895-04

**Authors:** Grant Molyneaux

**Affiliations:** Cincinnati, O.


### THE DENTAL REGISTER.

VOL. XLIX.] APRIL, 1895. [No. 4.

### COMMUNICATIONS.

### The Reason Why.

BY GRANT MOLYNEAUX, D.D S., CINCINNATI, O.

Read before the Ohio State Dental Society, December, 1894.

At the last meeting of this society, I had the honor of reading a paper entitled, "The Articulation of Artificial Teeth." In that paper I attempted to demonstrate that the utility of a denture depended largely upon a correct articulation of the teeth, and that this articulation should consist, not only of a nice adjustment of the cutting edges, but of a careful arrangement of the angles of the teeth with regard to the mechanical forces operative during mastication, and for the purpose of holding the dentures in position during use. However necessary the perfect arrangement of the teeth might be, it is not the only feature governing the successful construction of an artificial denture.

The teeth may be ever so perfectly articulated and the effect partially or totally lost if there is failure to perfectly adapt the dentures to the alveolar ridges and palate. In fact, we consider that the continued success of a denture depends quite as much upon the treatment of the model of the mouth, with a view of effecting a perfect adaptation, as upon any single feature connected with the construction of artificial dentures. By the proper treatment of the model, we mean the changing of the shape of the model, not in its entirety, but of certain portions that will insure at all times, and under all conditions, the maximum degree of retention possible in any given case.

The maximum degree of retention in superior dentures is not the same in all cases, nor is it possible to insure the same results in an unfavorable case that can be obtained in one where the natural advantages were considerable. But, with a full appreciation of the principles upon which the retention of dentures depends (barring mechanical means), and with the courage to carry them out to the fullest extent, many cases that by routine methods would be failures can be made to stay firmly in position. There are a great many mouths, the models of which require but little modification to effect an adaptation, while there are many more needing the most extensive alterations to obtain sufficient retention to support the denture, even during moderate use. It is with reference to these latter cases that our efforts are directed, hoping to present this subject in such a manner that the student will comprehend its object, and feel that in each case of artificial dentistry success depends upon individual study.

For a long time two theories have obtained regarding the force that holds superior dentures in place.

The first of these is the atmospheric pressure theory, and many are the adherents to this belief to-day. The atmosphere under usual conditions exerts itself in all directions simultaneously and its weight or pressure is not appreciated. When for any reason the air should be exhausted from a cavity, as for instance, a cartridge cap upon the tongue, the pressure of the air is noticed by the holding of the cap in position on the tongue. To appreciate the continuous pressure upon the atmosphere, we must be provided with an impervious cavity from which the air can be exhausted. A true illustration of this is the Madgeburg hemispheres, an experiment familiar to every school boy. When these hemispheres are placed together and the air exhausted, a force of more than fifteen pounds to the square inch of surface must be exerted to separate them, and they would remain intact indefinitely were not the air admitted by some mechanical means. If instead of one hemisphere we place over the opening of the other a piece of parchment or animal membrane, and attempt to exhaust the air beneath, we find that the membrane will not stand the pressure of the air and breaks. In order to avoid this breaking we place a column of water in contact with the membrane, and only partially exhaust the air. In a short time it will be seen that sufficient water has passed the membrane to equalize the condition beneath, and that the vacuum has been destroyed and that the membranous diaphram can be easily removed. Any other fluid would act the same as water, but the time required would depend on the density of the fluid. It is evident, that when the cavity beneath the membrane is filled with fluid, as it would be if a complete vacuum were produced, that although the membrane is held in place it is not by pressure of the air. If a partial vacuum be produced beneath animal membrane, it can only be for a short time, as one or two of three conditions will follow. Either air or moisture will pass through; or, the membrane, if sufficiently elastic and the cavity shallow, will fill the cavity completely, together with such fluids as may be in contact with the membrane. All of which is due to the elasticity and penetrable nature of animal tissue and the action of the atmosphere trying to establish an equilibrium.

The above has been used as an illustration because it is *parallel* to the condition that exists when "atmospheric pressure" dentures are inserted. If we cannot obtain a vacuum above our denture there could not possibly be a sensible pressure on its lingual surface to hold it in position.

When a denture containing the customary vacuum cavity on its palatine surface is inserted, the patient is told to close the mouth and swallow. This act is accompanied by a dilation of the thorax and closing of the posterior nares, which exhausts the air from the oral cavity underneath the plate as well. The air is still farther forced from under the plate by pressure of the tongue on the lingual surface of the plate. The tongue has no other exhausting power. The air is now nearly all removed from under our denture, and it is held for the time by atmosphere pressure partly. If the conditions would continue the same as immediately after insertion, there would be a constant support by the atmosphere, and the vacuum cavity would be the proper thing. Such however is not the case. If we allow our denture to remain in the mouth for an hour or so and then remove it, we will observe that the mucous membrane of the palate has been cupped down to almost fill the cavity in the plate, and that although the tissue covered by the plate was dry when the denture was inserted, that the plate is now covered with an oily fluid. It is evident now that our plate and mucous membrane are in contact throughout, save the intervening moisture, and that as all our space is obliterated we could no longer have atmospheric pressure if our illustration is correct. The question now presents itself, how is our denture supported if not by atmospheric pressure?

In an editorial review of Richardson's Mechanical Dentistry (last edition), one of the dental journals has the following to say:

"Discussing the force which retains a plate in position the author endeavors to show the 'absurdity of the atmospheric pressure theory, and maintains that it is necessary for retention by this means that a vacuum exist,' and then proceeds to annihilate the vacuum. We believe he is right in doing away with the vacuum, because if there be a vacuum it will soon be filled by loose tissue and it is evident it could not last long, but we will hold that it is the atmospheric pressure that secures the plate when he refers to it as the 'adhesion of contact.' *What is the adhesion of contact but the pressure of the atmosphere without a vacuum? It is the same pressure that forces water to follow the plunger up in the ordinary suction pump; that is, no vacuum that forces water up but the water rises to relieve a condition that would produce a vacuum.*" [Italics ours.]

The above quotation is given because it is the only argument that has been offered in support of the atmospheric pressure theory, and because it introduces a new phase of our subject. The question and seeming answer in italics are quite incongruous, as we know, that in order to have water rise in a suction pump, a removal of the air or pressure above the water must be accomplished, and that the water will continue to rise as the pressure is relieved until the column in the pump equals the pressure of the air when it will rise no higher, and to keep it there a vacuum must be maintained constantly above the column.

We also know that when two substances with perfectly flat surfaces are brought in contact, that some force is required to separate them; that this force is nothing like the force of the atmosphere; that in some instances it is only a few grains to the square inch, and in others more; that it varies with different substances; that if this force were due to atmospheric pressure it would be nearly constant; and finally, that this force is manifest in the absence of an atmospherc or in vacuum, and must be attributed to some power other than atmospheric pressure.

It is called "adhesion of contact," and certainly cannot be the same force as that which causes water to rise in the suction pump.

In any modern work of physics we see under the heading "Molecular Forces," three kinds of attraction, viz: Affinity, Cohesion, Adhesion.

"The Molecular attraction exerted between surfaces of bodies in contact is called adhesion, and is of three kinds."

Adhesion of solids to gases; Adhesion to solids to solids; Adhesion of solids to liquids.

It is this latter form of adhesion, that of solids to liquids, or rather solid and semi-solid with an intervening liquid, that claims our attention.

To have adhesion, two surfaces must be in contact, and it naturally follows that the more surface in contact the greater the adhesion.

The "adhesion between solids and liquids is greater than between solids." In the experiment of two plates of glass or two blocks of wood, with perfectly flat surfaces, when pressed together adhere with a certain force. But, it is impossible to perfectly adapt two hard surfaces mechanically to each other. If we place a film of water between in order to overcome the slight inequalities, we find the adhesion is very greatly increased. Continuing our experiment, we observe that the force required to separate the plates of glass or wood is greatest when exerted at right angles to the horizontal surface; when applied at other angles, the plates will slip or slide on each other and can be easily pulled apart.

Dr. Land, in his little book, "Scientific Adaptation of Dentures," makes a very practical application of this point.

This adhesion was formerly thought to be due to the pressure of the atmosphere, but as it can be thoroughly demonstrated in a vacuum it must be some other force and is "attributable to a reciprocal action between the surfaces of the bodies in contact." It is beyond all doubt the force of *adhesion* that maintains superior dentures in position, after the mucous membrane and plate are in contact. But, it is through the means of effecting a temporary pressure of the atmosphere, and through the elasticity of the mucous membrane that we can bring about a condition favorable to adhesion.

To further illustrate our point, we will ask the question: If the atmospheric pressure supports dentures, why is it that during febrile conditions when the mouth is dry or "parched" that the patient complains of looseness of the denture? Why is atmospheric pressure not operative now? Why is it that by painting the mucous membrane with a solution of alcohol and water or other stimulants that a perfect adhesion follows?

If we are satisfied that it is the force of adhesion that supports dentures, it behooves us to look carefully to every point that would lessen that adhesion, and improve every condition that would increase it. Adhesion, as we have stated, is the result of contact between the surfaces of bodies adapted to each other, but in the mouth adaptation means more than mere contact. An adaptation of two uniformly rigid bodies is at once and at all times the same, and pressure at any point on their horizontal surfaces would not change their relation.

In an artificial denture we have a uniformly rigid body. In the alveolar ridges and palate we have conditions anything but uniform. We find hard and soft spots arranged where they will be of least advantage. There is also the lower jaw playing over the superior denture at different angles, bearing harder on one point than another, the more compressible places giving way, while the plate rides on the harder points and loosens.

A denture may be ever so perfectly adapted for speaking purposes, but when the opposing teeth strike it at different angles, as in mastication, the plate will begin to tilt and loosen, which proves that an adaptation for talking is not necessarily one for masticating. We have seen plates tried for their "sticking" qualities by pulling on the anterior teeth. There is an exhibition of the force of adhesion under the most favorable circumstances, as all hard parts are relieved and the retention is that due to contact between mucous tissue and the plate. If pressure were exerted in the bicuspid region, or anteriorly, the denture might not stick so well.

Now, with a view to obtaining the greatest retention in superior dentures, one or all of four forms of treatment in the model may be necessary. First, treatment to relieve pressure on the hard parts, by thickening the tissues over these points.

The hard parts are usually found in the median line of the palate and over the ridge corresponding to the position of the two bicuspid and first molar teeth, called "buccal region." The tissue over the regions corresponding to the six anterior teeth and at the tuberosities is generally more compressible.

The amount of relief over the hard parts would be governed by the compressibility of the softer portions.

Second. Treatment to produce as much horizontal surface as possible.

Third. Treatment to restore the model to the size of the mouth, or as nearly as possible.

Fourth. Treatment by relief, to produce in extremely hard, flat or angular mouths a thickening of vascular tissue, over the entire palate for the purpose of obtaining more moisture, and a soft elastic padding for the denture to rest upon. Incidentally, reliefs are applied to models for the purpose of preventing pressure on points undergoing absorption, as well as points tender from surgical operation; also, to protect models from influences that might injure the model during the process of constructing a denture. In brief, we desire to obtain a uniform padding of vascular tissue under the denture that will prevent fulcrums and friction on the hard parts of the ridge and the consequent injury resulting from this continual irritation. Also, to obtain all the horizontal surface possible. The paper can be better understood by referring to some practical cases.

FIG. I

Fig. 1. This a flat mouth, all horizontal surface, and thickly padded with soft tissue over ridge, median line and entire surface of palate. There is nothing to be done to this model in way of reliefs, for there could be no improvement. A denture made for this case will be worn with unusual degree of satisfaction, providing there is a fair occlusion with opposing teeth. The only

FIG 2

treatment that we would suggest would be the tinning of the model with No. 40 foil to protect it in case of a vulcanite denture.

Fig. 2. This is also a flat mouth, representing more horizontal surface than Fig. 1. But in place of the soft vascular covering to the ridge and palate, we find the mucous membrane almost without color, it is so thin. It is also very dry, having a glazed appearance and the buccal and labial side ot ridge offers no assistance to support the denture, as this is angular.

This patient tried a number of operations but without good results. The plates when moistened would adhere fairly well for speaking, but even then would frequently drop. The treatment for this case according to our paper was to extend a relief over the entire palate of about 26-gauge, terminated at the junction of the palatine, with the labial and buccal surface of the ridge, and posteriorly at a line where the tissue was soft. The extent of this relief is shown in Fig. 2.

FIG. 2. B

Fig. 2 B. Is a vertical section of Fig. 2, and shows the relief B raised from the model.

The effect of this treatment was to bring positive contact at the circumference of the plate between the dotted line F, and to extend the plate as high as possible at the incisive and canine

FIG. 3.

fossæ and over each tuberosity. The space occupied by the relief B was soon filled with tissue and an excellent adhesion was the result.

FIG. 3A

Fig. 3. This is a model that first would appear simple, yet it was the source of much trouble to several operators. The trouble was not the lack of horizontal surface, but the uneven distribution of soft tissue and the resulting fulcrums. Anteriorly and posteriorly the mouth was soft, while in median line and buccal region the tissue was almost white it was so thin.

FIG. 3 B

The treatment of this case was to pad the three hard points by placing relief as indicated in Fig. 3A. As the space between the lateral and central relief in this case is very slight, and as the application of three reliefs would make several edges to irritate the tissue, we would suggest that the case be treated as in Fig. 3 B, with one relief covering the three points indicated in Fig. 3A.

Fig. 4. This is the verticle section of a V-shaped model, and will be recognized as one of the most difficult mouths in which to obtain a satisfactory degree of retention.

FIG. 4.

The manner of treating this model is shown in Fig. 4. The reliefs are adjusted ; first, to prevent pressure on the least compressible portions ; second, to create horizontal surface over the angles as shown by the shape of the reliefs in the diagram. By placing reliefs of uniform thickness, as shown in Fig. 4A, we

FIG. 4 A

prevent the pressure of the plate on these points, but we do not materially improve the conditions for adhesion as regards horizontal surface. The combined horizontal surface of E. E. C. Fig. 4, is quite that of the average mouth, and we have reason to believe, other conditions being equal, that a fair degree of retention will follow. These reliefs have been somewhat exaggerated in the drawing, it was not intended for them to appear quite so heavy.

Treatment to restore the model to the size of the mouth is advocated for vulcanite. The great trouble with vulcanite plates is that they are generally too small for the mouth, and that "misfits" are due to the fact that some mouths cannot be forced into the plate.

It is quite often that when a denture is first inserted that it will not readily go to place. The dentist helps matters along by vigorous stroking of the lingual surface, all of which is to crowd the mouth into a plate that is entirely too small for it. Such a denture cannot be worn without injury. From the time we take the impression, both by our own acts and those changes that take place in the materials with which we are working, we are getting farther away from the size and shape of the mouth, and a certain amount of alteration is necessary to prevent these changes from effecting the adaptation of the plate.

We have long advocated the use of No. 40 tinfoil over the models for vulcanite,and we know that if every vulcanite plate were made on a model tinned with No. 40 foil, that infinitely better results would follow than by the use of silex or collodion. When reliefs are to be placed on the model, they should be placed first, and the foil stenciled over them and the model.

The foil answers another purpose. During vulcanization when the rubber is expanding with great force, followed by its subsequent contraction and shrinkage, the model is protected from injury. (We cannot at this point refrain from recommending the careful study, by all those interested in vulcanite, of the papers by Dr. Geo. B. Snow in the *Dental Advertiser* of 1887.) The reliefs spoken of are formed of block tin and tinfoil, ranging from No. 40 foil to block tin of 20 and even 18 standard gauge, in thickness. They are placed on the model as indicated by an examination of the mouth. The edges are beveled and no abrupt margins left to irritate. The tissue thickened in this manner, after a time, becomes permanent and remains healthy.

We have never seen a case where the judicious use of reliefs ever produced harm. On the contrary, the patients who have experienced trouble appreciate to the highest degree this careful attention. It is not infrequent to find operators of years' experience claim they have never used "air-chambers," who at the same time tell how they shave the impression.

Now it does not make any difference whether you shave the impression or add to the model, the results are the same, though the object of the operators may be different.

We do not approve of the old-fashioned vacuum cavity with its sharp edges, as its only action is one of relief, to prevent pressure on the hard parts, and after these parts have been relieved let the edges be drawn out so that they are continuous with the model and not to be seen or felt. The form and thickness of reliefs are to be modified according to the material used in constructing the base of the denture, and to the conditions found in the mouth, and its shape.

A model for vulcanite or cast aluminum might require a little different and more extensive treatment than a model for a gold plate. A model for continuous gum would be different than either of the foregoing, but the principle involved in each case is the same.

We approve of adding to the model because the changes can be made more accurately and the reliefs can be blended off with the model so that their margin cannot be distinguished, and, consequently, mechanical irritation will not occur.

My apology for this paper is the number of cases I have seen by reference and consultation demonstrated that there are many not acquainted with the possibilities of prosthetic dentistry, and this is offered as a partial solution to "THE REASON WHY" of some of our failures.

DISCUSSION.

DR. AMES: I do not like to take the floor so often, being a visitor. The reading of Dr. Molyneaux's paper interested me very much, since the adaptation of dentures and the utilization of atmospheric pressure is one of my hobbies. Before the reading of the paper I was speculating as to what Dr. Molyneaux might have to show us, and I assure you it was a revelation to hear him tell that he could accomplish this change of form of the alveolar ridge by cupping down the tissues as he described. Of course we have all seen the tissues cupped by the ordinary air-chamber and become quite firm, but I must admit that to cushion up a plate, as he does, never occurred to me. There are some points, however, on which I must differ from Dr. Molyneaux, for he says that atmospheric pressure will be manifested without an air-space, and also that the utilization of atmospheric pressure is impracticable, because the tissues will either be drawn down or will have fluids drawn from them to defeat the purpose. This is practically the ground taken as I understood him. Now, I will not quarrel with any one about the capillary force and the adhesion of contact of moist surfaces, but what I want to claim is, that if one only utilizes these forces in the retention of artificial dentures, they do not accomplish the *maximum retention.* If a full upper denture is so constructed that it extends in all directions far enough, so that the entire periphery of the plate rests against and slightly displaces lax yielding tissue, we will not only get the adhesion of contact to retain the plate at ordinary times, but retention from atmospheric pressure at times when there is such pressure applied as would break the contact if the plate were not extended upon the lax, tissues at its entire periphery, by which means the air is prevented from readily passing beneath the plate as it would otherwise, and there is a *tendency* to the creation of a vacuum. There is not a *vacuum*, but a tendency to the creation of such a condition only, because the leverage is such that the entire or full atmospheric pressure of fifteen pounds to the square inch is not required to prevent the denture from coming away from the surface of the jaw. Dr. Molyneaux rightly states that the tissues will not tolerate a vacuum. While under the conditions ordinarily met with in connection with the wearing of dentures there is not sufficient traction brought upon the tissues, when the denture tends to leave them, to cause rupture of the capillaries. I have repeatedly caused the rupture of the capillaries of healthy tissue during demonstrations of this principle, by means of a denture having attached to it, near the second molar of each side, a strong twine, by means of which sufficient traction could be brought upon the tissues by a *tendency* to the creation of a vacuum to cause a rupture of the capillaries, sufficient blood flowing therefrom to admit a breaking of the joint at the edge. The Magdeburg hemispheres I do not consider an illustration of what can or cannot be accomplished in the mouth. The illustration does not fit the case. I see where a great deal of good can be accomplished by the system of relief so ably brought out by Dr. Molyneaux, and I am very grateful to him for the points I have gotten in this line. With this cushioning up of such angular jaws and the carrying of the plate to the soft tissues in all directions, the maximum of retention will be such as to surprise most operators. To carry out the plans I am advocating a very carefully taken impression is called for. If the patient has an old denture I generally use this as the tray, extending it back with wax, if necessary, to get an impression of sufficient surface. If there is not an old denture available, an impression is taken in the ordinary way, a model made, and over this a blank or base-plate is formed, using the " Ideal Base-Plate." Trim the edges of this so that it does not press against and distend any of the tissues of the lip or cheeks and extends a little farther upon the soft palate than it is desired to extend the plate, which is to simply reach to the soft palate. This base-plate used as an impression tray, using soft plaster, should give an impression without putting any of the tissues upon tension, *i. e.*, without stretching any of the soft parts out of the natural position. By making a model from such an impression the rim of the plate can be made to nicely fill in all space between the jaw and the lip and cheeks, so that no air can enter at part of the periphery of the plate and by grooving across the model from a point outside one tuberosity to the same point on the opposite side, following the line of the beginning of laxity of tissue, the joint will be complete throughout the entire periphery. A very large majority of mouths will tolerate such a denture comfortably. In an occasional rare case other and more complicate means must be resorted to for accomplishing this satisfactory retention.

DR. TAFT: I have here a denture that some of those present have seen. It has the following history: A person who had lost all her teeth from the lower jaw desired to have an artificial denture. There was no dentist within that region. She took a piece of 16 to 18 wire and bent it as near as she could to the size and shape of the arch from which the teeth had been taken. She procured the teeth of some animal, probably a sheep, and ground them, as she supposed, to about the size they ought to be —ground them on a grindstone; used twine, winding it about wire and attached the teeth, having ground out of the lower end a groove to enable the twine to hold them; she wound it around the teeth until they were firmly held in position. This piece was worn about three years and did service all that time. After this a dentist came along and she then had a set made by him.

I obtained it from the son of the woman who made and wore it. So sensitive was she about it that her family knew nothing of it till it was in the mouth and she was using it. I mention this to show that uncouth things can be worn in the mouth and for a long time. It is almost incredible, but it was stated positively by a son of the person who made and wore them.

We have difficulty sometimes with well-fitting plates to induce people to wear them. I have occasionally shown this to such persons with good effect, and usually it makes people wonder why they can not wear a well-fitting plate. It illustrates the proverb, "where there is a will there is a way."

Now, a word in regard to bridge-work. It is quite evident that from the expression here, a reaction is taking place in respect to the use of bridge-work. We have often been annoyed at the excess to which that kind of dentistry has been carried. There are dentists who make that almost an exclusive practice. Does not a large proportion of this work fail within one to three years after it is introduced? Occasionally pieces are found put in a number of years ago that seem to be doing well yet, but a large proportion of them show a failure within one to three years, and it is well this reaction should take place. It makes one indignant to find solid, living teeth cut away and ruined to introduce a piece of bridge-work. It is little short of an outrage upon humanity. I want to offer this protest against the general and wholesale use of bridge and crown-work by so many of the profession. It is a very profitable kind of work. The charges usually made are far more than for ordinary plate work. Cupidity comes in here, and it ought to be ruled out.

Dr. G. L. Field, of Detroit: I am glad to hear Dr. Taft make this little speech here. I feel the same way he has expressed himself, feeling indignant at the work done by men who ought to have known better who put in bridge-work only to make dollars and cents out of it, and this will not, only in exceptional cases, last but a short time until it begins to give way. I saw a case, in a college I am connected with in Detroit, that made me extremely indignant, because it was in my own department, where we should have known about it. One student undertook to make a piece of bridge-work for another, and I happened to see it. One of the men at one chair said, "I am busy." I said, "What have you to do?" He said he had a piece of bridge-work to put on Mr. M., one of the students. He was trying to replace a molar that had been removed in childhood. He had ground away a molar and bicuspid that were perfectly sound to put in a poor-good-for-nothing bridge.

Dr. Molyneaux, in closing the discussion, said that the models used to illustrate the paper were prepared especially for vulcanite work, but that the principles were involved in all kinds of dentures, but modified to suit the material and method used. Vulcanite work has done more to ruin mouths than anything else, not so much on account of the vulcanite, but the careless methods of using it. Vulcanite is an excellent base when properly made. I believe that much of the bad results from vulcanite are due to the interference to nutrition which follows the insertion of many vulcanite plates made directly over a plaster model. The plate is too small for the mouth, and the tissues must be cramped in order to get the denture in place. Another trouble, "rubber sore mouth," I have often seen corrected by simply making a vulcanite plate with self-cleansing surface, such as is obtained by vulcanizing against thick tinfoil.

Dr. ——: Suppose you show us how you put your tinfoil on the model.

Dr. Molyneaux: If the society wants to be detained a little longer, I will show that for the benefit of those who might not understand it. Putting on the tinfoil is a very simple process. I have been accustomed to use both Ashmead's and Nye's tinfoil. I suppose any other would do as well. Take any form of model you like and a number forty tinfoil or number sixty. You put reliefs on the model wherever indicated. You take shellac, of the consistency of thick syrup, and cover the model, or that part of the model you want the plate to cover; then you take your piece of tinfoil, one sheet, that is Number 40, laying it over the model. Take a stencil brush, with quick successive taps and stencil the foil until every portion is in contact with the model, then with a piece of chamois rub over a couple of times until smooth. If you use a burnisher you can not get it down for a long time, and also get it full of wrinkles. You trim off the excess and then take your burnisher and go around the edge and if there be any wrinkle, as there might be, rub it down, and if you leave any impression of the stencil brush, the rubbing with chamois brings it down perfectly to the model, and you have a smooth surface. If you use a thinner tinfoil than forty it does not act as any protection to the model at all. Putting your shellac on first and then taking your sheet of tinfoil and going down over the edges in that way, you see it is over there perfectly smooth, and by rubbing it down with a piece of linen or something you have handy, it gives a surface on which you can vulcanize. The use of the stencil brush is an important thing. If you use thin shellac it will not hold the foil.

## Figures and Tables

**Fig. 1 f1:**
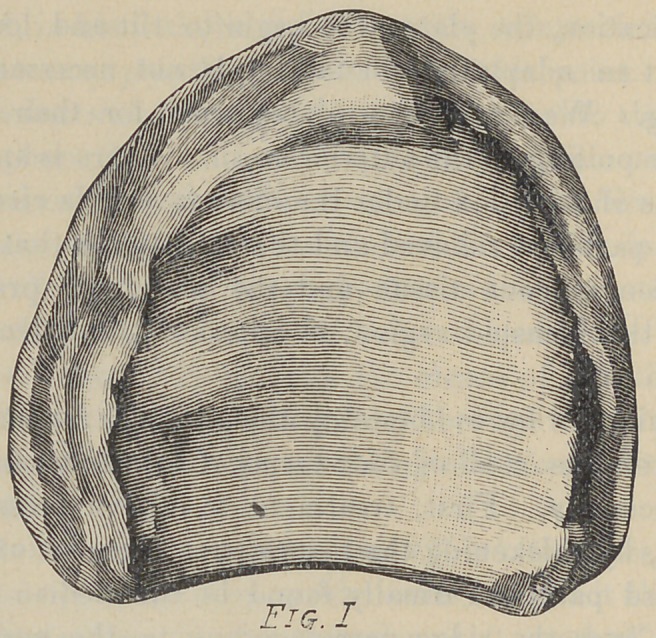


**Fig. 2 f2:**
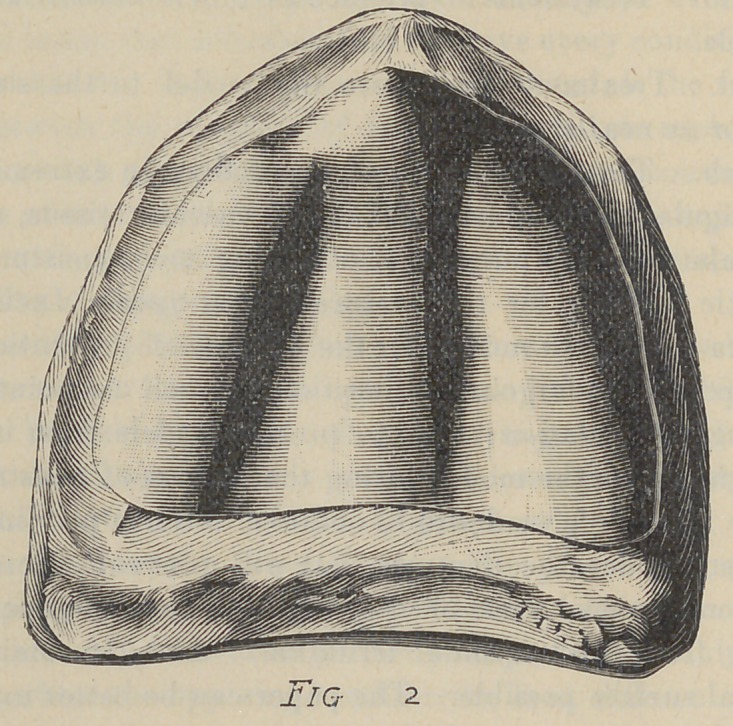


**Fig. 2B f3:**
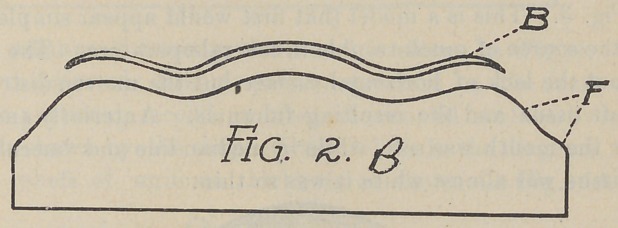


**Fig. 3 f4:**
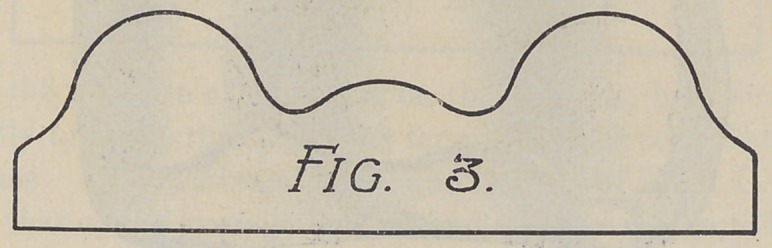


**Fig. 3A f5:**
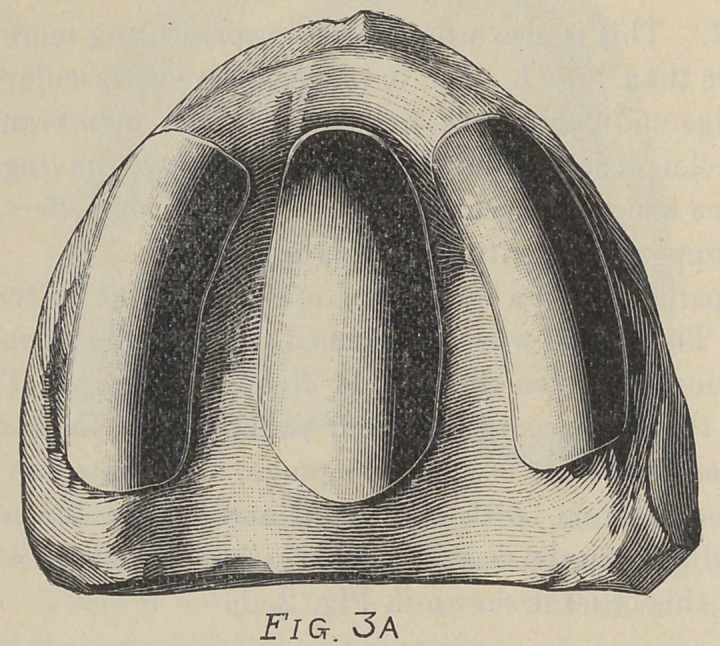


**Fig. 3B f6:**
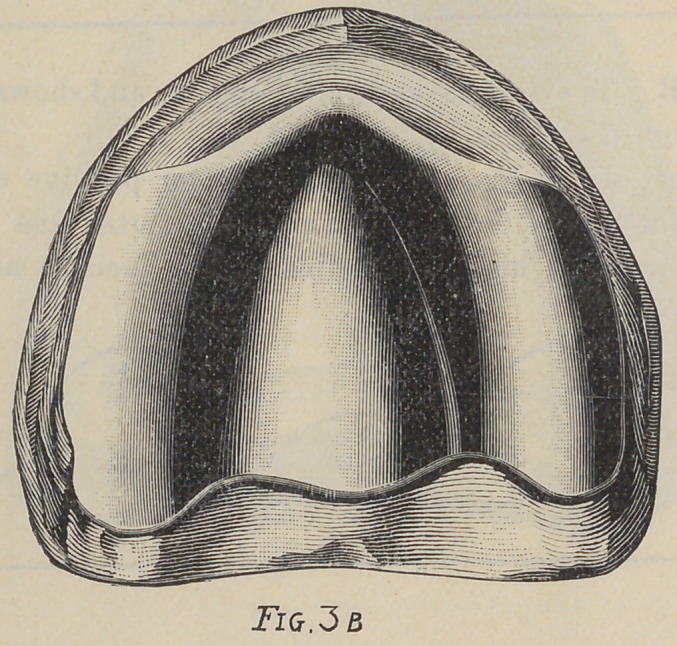


**Fig. 4 f7:**
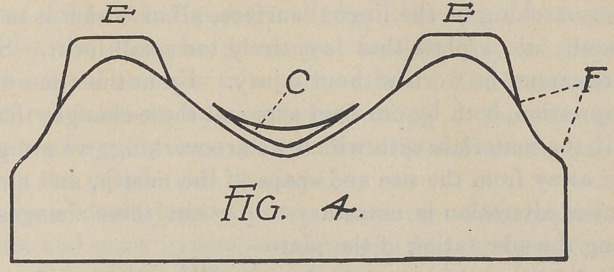


**Fig. 4A f8:**